# Opportunistic complexes of *E. coli* L-asparaginases with citrate anions

**DOI:** 10.1038/s41598-019-46432-0

**Published:** 2019-07-30

**Authors:** Jacek Lubkowski, Waikin Chan, Alexander Wlodawer

**Affiliations:** 1 Macromolecular Crystallography Laboratory, Center for Cancer Research, National Cancer Institute, Frederick, MD 21702 USA; 2 Department of Bioinformatics and Computational Biology and The Proteomics and Metabolomics Core Facility, The University of Texas MD Anderson Cancer Center, Houston, TX 77030 USA

**Keywords:** X-ray crystallography, Paediatric cancer, Hydrolases

## Abstract

Active sites of enzymes are highly optimized for interactions with specific substrates, thus binding of opportunistic ligands is usually observed only in the absence of native substrates or products. However, during growth of crystals required for structure determination enzymes are often exposed to conditions significantly divergent from the native ones, leading to binding of unexpected ligands to active sites even in the presence of substrates. Failing to recognize this possibility may lead to incorrect interpretation of experimental results and to faulty conclusions. Here, we present several examples of binding of a citrate anion to the active sites of *E. coli* L-asparaginases I and II, even in the presence of the native substrate, L-Asn. A part of this report focuses on a comprehensive re-interpretation of structural results published previously for complexes of type I L-asparaginase (EcAI) from *E. coli*. In two re-refined structures a citrate anion forms an acyl-enzyme reaction intermediate with the catalytic threonine. These results emphasize the importance of careful and critical analysis during interpretation of crystallographic data.

## Introduction

Over the last several decades macromolecular X-ray crystallography^1^ has been utilized to illuminate molecular properties of biomolecules and their systems. Crystal structures enabled understanding of the inner workings of enzymes, receptors, transporters, etc., and provided the most detailed description of molecular machines. However, a major limitation of this technique is the necessity to prepare diffraction-quality crystals of the material under study. Crystallization protocols rarely employ conditions mimicking the natural environment of target macromolecules. In most cases concentration of the protein is much higher than biologically relevant and the process of crystallization is assisted by additional components, such as buffers, precipitating and dehydrating agents, additives, co-factors, etc. Such additional factors may affect the results and an evaluation of their influence may be necessary.

The citrate anion plays a significant role in macromolecular crystallography, as various citrate salts are components of crystallization screens. This is due to the properties of citric acid, which combines in a comparatively small molecule three carboxylate groups and one hydroxyl. Therefore, owing to evenly-spaced pK_a_ values of the carboxylate groups (3.13, 4.76, and 6.40)^2^, citrate is a component of buffers that are effective over a wide pH range (3–6.5). Additionally, citrates are also utilized as additives in crystallization solutions. Citrate ions are present in commercial crystallization screens at concentrations varying from 100 to over 200 mM, i.e. two to three orders of magnitude higher than those of crystallized biomolecules^3^. While high concentration of a component of crystallization solution is not unusual (and often is required), under some circumstances this may pose a challenge during interpretation of the results.

We identified a case of such a problem during the interpretation of crystal structures L-asparaginase. L-asparaginases catalyze the hydrolysis of L-Asn to L-Asp. The most extensively studied are the type II L-asparaginases from *Escherichia coli* (EcAII) and *Erwinia chrysanthemi* (ErA). These enzymes have been successfully used for over 40 years in the treatment of selected leukemias and lymphomas^4–7^. The use of the abovementioned L-asparaginases is governed primarily by their therapeutic efficiency, economic factors, and the familiarity gained over many years. However, many other potential sources of L-asparaginase have been identified, since a majority of bacteria carry genes encoding at least one L-asparaginase. The genome of *E. coli* encodes three L-asparaginases, called type I, II, and isoaspartyl aminopeptidase/L-asparaginase, respectively^8^. EcAI and EcAII are structurally closely related, with their respective amino acid sequence identity of 25% and additional similarity of 20%. The active sites of these enzymes are also very similar, both in their composition and in the structural arrangement. The main difference, however, stems from their substrate affinities, with the K_m_ for EcAII over hundred times higher than for EcAI.

Although L-asparaginase has been quite successfully used as a drug^9^, its utilization has not been free of complications. The major side effects of L-asparaginase therapy are attributed to non-specific activity of the enzyme (i.e. a minor L-glutaminase activity), immunogenic properties of a bacterial protein, and limited *in vivo* stability of the enzyme. Thus continuous efforts to improve these therapeutic preparations are ongoing, focused on better understanding of the molecular basis of the activity and specificity of the enzyme^10–12^, as well as on inventing novel ways of therapeutic application^9^. While performing crystallographic studies of EcAII as part of such efforts, we frequently observed citrate anions located in the enzyme active site.

Since we were interested in studying the intermediate states of the catalytic process, during a review of previously published results we encountered a report describing a covalent intermediate in the active site of EcAI (AnsA) crystallized from citrate buffer^13^. That study was aimed at elucidation of the structural principles of the allosteric regulation of that enzyme. Yun *et al*. determined two structures of the ligand-bound enzyme (Protein Data Bank (PDB) entries 2p2n for the wild type (wt) EcAI and 2him for the T162A mutant). The mutated residue is located in the immediate vicinity of the putative allosteric site, but quite distant from the substrate-binding site. In the structurally very similar EcAII the active site was commonly observed to be occupied by non-covalently or covalently bound substrate^14,15^. Yun *et al*. reported covalent binding of the substrate (presumed to be L-Asn) to Thr14 (equivalent to Thr12 in EcAII), the residue previously implicated as a possible nucleophile in the first step of the catalytic deamidation of L-Asn^14,16^. The only previous description of a similar modification of the active site of L- asparaginase was presented in a report of the structure of the T89V mutant of EcAII^15^.

Since our own extensive crystallization efforts that utilized EcAII(wt) and its mutants under a wide range of conditions often resulted in structures in which bound citrate was observed, we became interested in the mode of binding of this opportunistic ligand. In all cases only non-covalent mode of binding of citrate to EcAII was observed and we show here that such binding is strongly influenced by the conditions under which an experiment is performed, potentially influencing the binding of the usual substrates or products. The finding of citrate bound to EcAII led us to analysis and, subsequently, reinterpretation of the results published by Yun *et al*. for EcAI. We concluded that these authors may have downplayed the possibility that the ligand reported by them to be an acyl-enzyme intermediate of the conversion of L-Asn to L-Asp could actually be covalently bound citrate.

## Results

In order to simplify the descriptions that follow, we employed three conventions applicable to all type I and II L-asparaginases. The active site pockets of these enzymes are shaped by two protomers, one of which forms most of the active site scaffold, including all catalytic residues; it will be referred to as a “major” protomer, with the second protomer called “minor”. Native substrates of L-asparaginases are L-Asn and, to a limited extent, L-Gln, although β-protonated L-Asp (and presumably β-protonated L-Glu) are also processed by the catalytic machinery of these enzymes^17^. All substrates bind to the active sites of L-asparaginases according to a common scheme, thus they will be referred to as canonical ligands that form canonical complexes with L-asparaginases through canonical interactions. An example of such a canonical complex is illustrated in Fig. S1, which depicts details of the interactions between L-Asp and EcAII (PDB ID 3eca)^14^. In a canonical complex, residues Thr12, Tyr25, and Val27, all placed within the flexible hairpin located near the N terminus, accommodate a unique conformation (depicted in Fig. S1), usually referred to as “closed conformation”. In the absence of a canonical ligand, a significant part of the N-terminal hairpin is disordered and is often referred to as being in an “open conformation” or, more accurately, an “open state”.

### Structures of EcAI (wt and T162A) at pH 4.0

These high-resolution structures were re-refined using diffraction data deposited in the PDB. No significant changes needed to be made to the bulk of the coordinates of the four molecules found in the asymmetric unit. Some external loops of the protein, that were not modeled during the original refinement, were left unmodeled, although a few additional residues could be added. The ligand molecules in the allosteric sites were seen very clearly in the corresponding electron density, although their identity (L-Asp *vs*. L-Asn) could not be determined. A number of water molecules with very low atomic displacement parameters (ADPs), located near basic groups, were replaced by chloride ions (present at a very high concentration in the crystallization buffer) and some clusters of water molecules, originally modeled too close to each other, were re-interpreted as ethylene glycol. No attempt was made to identify every remaining peak in the difference density and thus some solvent molecules might still not have been placed. The overall quality of the original models was satisfactory (Table 1) and, while the re-refined models had a slightly better MolProbity^18^ clash scores, lower R-factors, and comparable total scores, improvements were modest and may have been due in part to the differences in the refinement software and procedures (Table 1).

**Table 1:** Selected parameters of the originally deposited (left column) and re-refined (right column) structures of EcAI complexes.

Protein	EcAI(wt)		EcAI(T162A)	
PDB accession code	2p2n (original submission)	6nxd (re-interpreted)	2him (original submission)	6nxc (re-interpreted)
Resolution [Å]	50.0–1.90 (1.95–1.90)	45.17–1.90 (1.95–1.90)	50.0–1.82 (1.82–1.89)	46.54–1.74 (1.79–1.74)
No. of reflections total/*R*_*free*_	94487/4744	94513/4749	115053/5828	126795/6415
Completeness [%]	90.7 (81.3)	90.7 (81.3)	96.7 (89.1)	93.9 (67.4)
*R*/*R*_*free*_ [%]	22.3/26.4	20.5/24.4	20.9/22.6	16.8/20.8
Protomers in ASU	4			
ADP parametrization	Isotropic			
No. atoms				
Protein	9696	9701	10013	10148
Ligands	52	146	136	145
Water	575	564	515	564
Ramachandran statistics [%]				
Favored/outliers	97.9/0.2	96.4/0.5	96.6/0.7	96.6/0.5
R.m.s. deviations from target values				
Bond lengths [Å]	0.013	0.010	0.005	0.019
Bond angles [°]	1.37	1.70	1.4	2.26
Clashscore/percentile	7.36/90^th^	6.71/93^rd^	11.5/66^th^	5.58/92^nd^
MolProbity score/percentile	1.97/70^th^	2.01/67^th^	1.93/69^th^	1.85/69^th^

**Figure 1:**
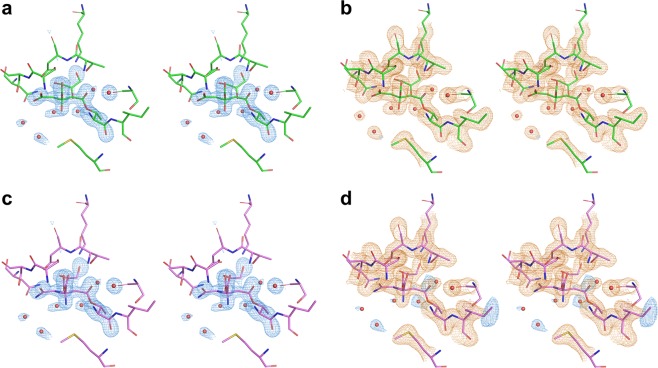
The active site region of the EcAI(T162A). Protein residues are shown as sticks, the 2mFo-DFc maps contoured at 1·σ level are shown in orange, mFo-DFc maps contoured at 3·σ level are blue, with no significant negative density present at −3·σ level. (**a**) Omit map (mFo-DFc) for the re-refined model (PDB ID 6nxc) with Thr14, citrate, and several solvent molecules removed from phase calculations, superimposed on the final model. (**b**) The final 2mFo-DFc map for the re-refined structure, with no difference density remaining at +*/*−3·σ level. (**c**) Omit map for the original structure (PDB ID 2 him), calculated as in panel a), with the original coordinates superimposed. (**d**) The final maps calculated for the originally deposited model. A significant positive density is present in the difference map.

**Figure 2:**
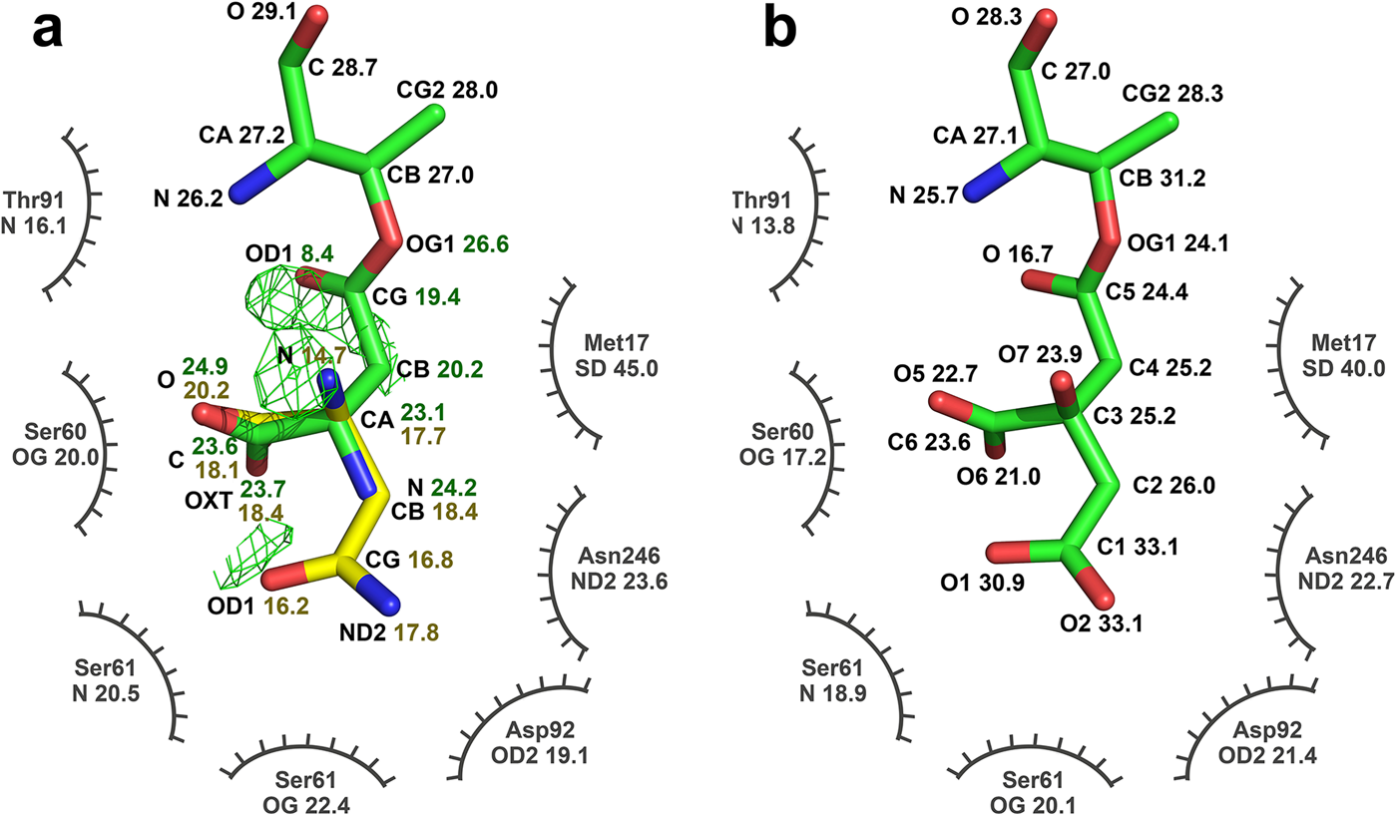
Two interpretations of the structure of Thr14 and its ligand. The mFo-DFc electron density map contoured at +3σ is shown in thin green lines. Values of ADPs of the individual atoms of ligands and those contributed by nearest residues of the enzyme are marked next to their symbols. (**a**) In the original structure (PDB ID 2him) Thr14 and the covalently bound L-Asp are shown in green sticks, whereas the partially overlaid L-Asn is yellow. (**b**) Re-interpreted structure (6nxc) is labeled in an analogous way. No residual density is present at the +3σ level. In both panels, oxygen and nitrogen atoms are colored in red and blue, respectively.

A major difference in the re-interpreted structures was substitution by citrate anions covalently linked to the side chain of Thr14 through ester bonds of the superimposed, partially occupied L-Asp/L-Asn. Both the original model and the re-refined model appear to fit the mFo-DFc (where Fo and Fc are the experimentally measured and model-based structure amplitudes, respectively, m is the figure of merit, and D is the sigma-A weighting factor) omit maps quite well (Fig. 1a,c). Substitution of the ligands did not result in significant changes to the 2mFo-DFc electron density maps for this region (Fig. 1b,d), but resulted in elimination of residual (and originally unexplained) peaks in the mFo-DFc difference map (Fig. 1b,d2). As seen in Fig. 2, the ADPs of the atoms contributed by the citrate anion became much more uniform and comparable to the ADPs of their environment, being lowest in the vicinity of the covalent bond with the enzyme and just slightly elevated for the distal parts of the ligand. The uniform distribution of the ADPs and the lack of features in the difference mFo-DFc electron density maps provide strong support for this interpretation of the identity of the ligand. It should be pointed out that the citrate carboxylate group distant from Thr14 is H-bonded to the amide nitrogen and the side chain hydroxyl of Ser61, serving as an H-acceptor regardless of the protonation state of this carboxylate (this group was originally modeled as the side chain carboxamide of L-Asn).

The structure of the native EcAI was refined by Yun *et al*. with Refmac5 and a new version of the same program was used for the current re-refinement. Since only minimal changes were made to the re-refined coordinates, the lowering of the R-factors (Table 1) may have been mostly due to the improvements of the software. Whereas the original model was missing part of the N-terminal hairpins consisting of residues 14–39 (with Thr14 modeled only in molecule A), we found that even the starting maps had some indications of rather weak density for a number of missing residues, indicating high flexibility. Although we modeled Thr14 with a covalently bound citrate anion in all four protomers, the corresponding mFo-DFc map for this residue and its ligand is completely featureless (indicating a very good fit) only for protomers A and B, each belonging to a different dimer of EcAI. The electron density was less contiguous in the case of protomers C and D, suggesting that Thr14 is more flexible in one protomer in each of the two dimers than in the other one. This result differs very significantly from the original interpretation in which Thr14 was not modeled in molecule B. With the ADPs of citrate anion being somewhat lower than those of Thr14, we assume that the citrate anion is bound in the same fashion regardless of whether it is covalently bound to Thr14 or not, and that partial disorder of the protein chain in the vicinity of Thr14 led to the increased ADPs. However, we did not attempt to refine such an alternative model.

The structures of both the native enzyme and the T162A mutant are very similar near the mutation site, with the only consistent difference being the presence of a water molecule located between Thr14 OG1 and Gln272 O in the native enzyme, but not in the mutant. All other water molecules located in the vicinity of the mutation site are practically the same in both structures.

### Structures of EcAII(wt or D90T, K162T) crystallized from citrate buffer at various pHs

In our studies of EcAII and its mutants we observed that these proteins can readily crystallize at various pH from mixtures of the enzyme solution and equivalent volumes of precipitant solution containing 17% (w/v) PEG3350 and 0.17 M ammonium citrate. The structure denoted EcAII^wt^-apo7 describes crystals of the EcAII(wt) grown at pH 7. These crystals belong to the space group C2 and the diffraction data collected at 100 K extended to the resolution of 1.75 Å. There are four protomers in the asymmetric unit, organized in two dimers, each representing half of the biological assembly. The original goal of that experiment was structural characterization of a ligand-free active site of EcAII. Two structures of EcAII are deposited in the PDB (3eca and 1nns), but in both cases the active sites of the enzyme are occupied by the canonical ligand, L-Asp. An additional structure (PDB ID 6eok) represents the PEGylated EcAII^19^. Unfortunately, we did not achieve an intended goal. The 2mFo-DFc electron density peaks indicated the presence of a large ligand, equivalently positioned in each of the four active site pockets (see Fig. S2a). The shape of this peak could be unambiguously interpreted as a citrate anion. Details of the interactions stabilizing citrate binding are discussed in a separate section. The overall fold of EcAII^wt^-apo7 is the same as that of the canonical complex (PDB ID 3eca). Two notable conformational changes are found, however, in the active site cavity. The first is the reorientation of the Glu283 side chain, contributed by the minor protomer, away from the negatively-charged citrate ligand. Secondly, the N-terminal hairpin (residues 11–35) is mostly disordered and not modeled. The ordered fragments of this hairpin vary in four protomers, due to different stabilization by crystal contacts, but positions of Thr12 indicate in all cases the presence of the open state of this motif, which is in contrast with the canonical complex, where the flexible hairpin assumes a unique closed conformation.

Three structures included in this report, referred to as EcAII^D90T/K162T^-L-Asn50, EcAII^D90T/K162T^-L-Asn56, and EcAII^D90T/K162T^-L-Asn62, were determined for crystals of the EcAII^D90T,K162T^ variant grown in the presence of 10 mM L-Asn at pH 5, 5.6 and 6.2, respectively. Although equivalent structures have been also determined at pH’s 5.2, 5.4, 5.8, and 6.0, due to their redundancy they are not presented here. Crystallization under the same conditions was unsuccessful at pH higher than 6.2. None of crystals from this group could be successfully frozen, as all attempts led to dramatic decrease in the quality of diffraction, both in terms of resolution and mosaicity. Therefore, diffraction data were collected at room temperature. All three structures, refined at the resolution of 2.15 Å or higher (Table 2), are isomorphous and belong to the space group P3_1_21 with two protomers, representing half of the biological assembly, present in the asymmetric unit. We found that the structures are practically invariant within the pH range explored in this study, both in terms of the overall fold as well as details within the active site region. Therefore, we will describe only the structure of EcAII^D90T/K162T^-L-Asn50. Somewhat unexpectedly, we found that in all three structures one of two independent active sites (in protomer B) was occupied by a citrate anion, despite the fact that L-Asn was present in the crystallization buffer, which is illustrated in Fig. S2c. The position of the citrate in these structures differs significantly from those observed for EcAII^wt^-apo7 or for complexes of EcAI. In this case the ligand is stabilized by interactions with the affinity His_6_-tag, contributed by a symmetry-related protomer, therefore the observed binding mode is an experimental artifact. Despite this difference, however, there is one common denominator, namely in all cases discussed so far (including the canonical complex) the interaction of a negatively charged α-carboxyl group of citrate with Ser58 of EcAII (or with equivalent Ser60 in EcAI) is invariant. Compared to the structures of wild type L-asparaginases, mutation to threonine of two key active site residues, Asp90 and Lys162, significantly changes the network of interactions in the active site pocket. An invariant set of hydrogen bonds linking the active site triad (Asp90-Lys162-Thr89) does not exists in EcAII^D90T/K162T^; however, orientation of the side chain of the retained Thr89 remains the same. Similarly to the structure EcAII^wt^-apo7, the side chain of Glu283 (from the minor protomer) is reoriented compared to the canonical complex and the N-terminal hairpin is largely disordered. While the active site appears unoccupied in protomer A, there is a blob of the mFo-DFc electron density adjacent to the side chain of Ser58. Its size, triangular shape and particular location suggest that this peak may represent a carboxylate moiety. Such interpretation postulates that either L-Asn or citrate are anchored to the active site through their α-carboxyl group, with the remainder of either ligand disordered. Since we cannot unambiguously interpret this electron density, for the purpose of refinement we modeled it as an acetate anion. When refined, position of the acetate aligns very well with the α-carboxyl groups of citrates, discussed earlier. Conformations of the residues in active sites A and B are very similar.

**Table 2:** Diffraction data collection and processing for the EcAII crystals.

	EcAII^D90T/K162T^-L-Asn50	EcAII^D90T/K162T^-L-Asn56	EcAII^D90T/K162T^-L-Asn62
Crystal-to-detector distance (mm)	75	75	60
Rotation range per image (°)	0.75	0.75	0.75
Total rotation range (°)	75	77.25	90
Exposure time per image (s)	200	200	240
Temperature (K)	293	293	293
Space group	P3_1_21	P3_1_21	P3_1_21
*a, b, c* (Å)	126.17, 126.17, 89.20	126.20, 126.20, 89.44	126.28,126.28,89.69
*α, β, γ* (°)	90, 90, 120	90, 90, 120	90, 90, 120
Mosaicity range (°)	0.41–0.55	0.38–0.40	0.38–0.41
Resolution range (Å)	2.15–40.0 (2.15–2.19)*	2.15–40.0 (2.15–2.19)	1.85–40.0 (1.85–1.88)
Total No. of reflections	180,060	168,511	333,956
No. of unique reflections	43,555	44,670	70,498
Completeness (%)	97.1 (99.8)	99.3 (99.5)	99.9 (99.7)
Multiplicity	4.13	3.77	4.74
<I/σ(I)>	12.48 (1.79)	12.08 (2.14)	22.84 (2.03)
*R*_merge_^†^	0.108 (0.686)	0.102 (0.632)	0.071 (0.750)
*R*_r.i.m_^‡^	0.059 (0.442)	0.059 (0.385)	0.036 (0.420)
B from Wilson plot (Å^2^)	22.2	22.4	18
CC_1/2_	0.880 (0.518)	0.910 (0.672)	0.948 (0.737)
	**EcAII**^**wt**^**-apo7**	**EcAII**^**D90T/K162T**^**-L-Asn7**_**S**_	**EcAII**^**D90T/K162T**^**-apo7**
Crystal-to-detector distance (mm)	60	75	75
Rotation range per image (°)	0.5	0.3	0.5
Total rotation range (°)	180	180	180
Exposure time per image (s)	220	230	240
Temperature (K)	100	100	100
Space group	C2	C2	C2
*a, b, c* (Å)	151.35, 62.35, 142.67	152.01, 62.54, 143.24	151.89, 62.50, 143.16
*α, β, γ* (°)	90, 118.0, 90	90, 118.2, 90	90, 118.2, 90
Mosaicity range (°)	0.64–0.69	0.45–0.48	0.42–0.47
Resolution range (Å)	1.75–40.0 (1.75–1.78)	1.97–40.0 (1.97–2.00)	1.93–40.0 (1.93–1.96)
Total No. of reflections	336,615	263,837	284,693
No. of unique reflections	116,189	81,136	86,035
Completeness (%)	98.1 (96.1)	96.6 (92.1)	96.1 (79.0)
Multiplicity	2.9 (2.4)	3.3 (2.6)	3.3 (2.5)
<I/σ(I)>	21.60 (1.99)	11.3 (1.86)	11.50 (1.92)
*R*_merge_^†^	0.039 (0.405)	0.095 (0.511)	0.100 (0.512)
*R*_r.i.m_^‡^	0.026 (0.292)	0.061 (0.365)	0.064 (0.359)
B from Wilson plot (Å^2^)	14.6	22.3	12.4
CC_1/2_	0.950 (0.769)	0.911 (0.674)	0.907 (0.646)

^†^R_merge_ = Σ(|(I − <I>)|/Σ(I). ^‡^Estimated R_r.i.m._ = R_merge_[N/(N − 1)]1/2, where N is the data multiplicity.

*Values shown in parentheses correspond to the highest resolution shell.

The remaining two structures included in this report were determined for crystals of EcAII^D90T/K162T^ grown under the same conditions as EcAII^wt^-apo7, discussed earlier. In the first case, EcAII^D90T/K162T^-apo7, diffraction data were collected from a crystal first transferred to the cryoprotecting solution and subsequently frozen at 100 K. For the second structure, EcAII^D90T/K162T^-Asn7s, a crystal was soaked for several minutes prior to freezing in the cryoprotecting solution containing 10 mM L-Asn. Both structures are isomorphous with EcAII^wt^-apo7 in space group C2. The active sites are very similar for all protomers in both structures, as well as similar to protomer A in EcAII^D90T/K162T^-L-Asn50, described earlier. Minor differences apply to a distribution of solvent in the active site cavities and to the extent of disorder of the N-terminal hairpin. A blob of electron density was found in all active sites adjacent to the side chain of Ser58. While in the case of EcAII^D90T/K162T^-apo7 this density is most likely attributed to the α-carboxyl group of citrate, in the case of EcAII^D90T/K162T^-L-Asn50 the origin of the electron density remains uncertain. We likewise modeled it as an acetate anion.

### Binding of citrate to the active sites of *E. coli* L-asparaginases

**Figure 3:**
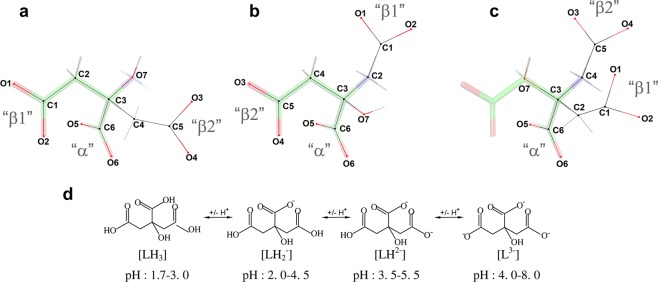
Mimicry of L-aspartate anions by citrate. A molecule of L-Asp is shown in a stick representation, in the conformation found for the complex with EcAII (PDB ID 3eca, monomer A). The overlaid citrate anions, drawn in line representation, are shown in three conformations, in which the α-carboxyl groups of L-Asp and citrate are aligned. Non-H atoms of citrate are labeled according to a convention used in crystal structures. Each of the three carboxylate groups (in citrate anion) is uniquely labeled, as α, β1, or β2 (panels a–c). While it appears that conformations of citrate shown in panels a and b fit the L-Asp best, mimicries depicted in panels b and c were observed in the structures described in this study (**b** in the case of re-refined entry 2him, new PDB ID 6nxc; **c** in the case of EcAII^wt^-apo7, PDB ID 6nxb). Panel d illustrates protonation states of citrate at different pH values; it is generated based on data by Rao *et al*.^35^.

**Figure 4:**
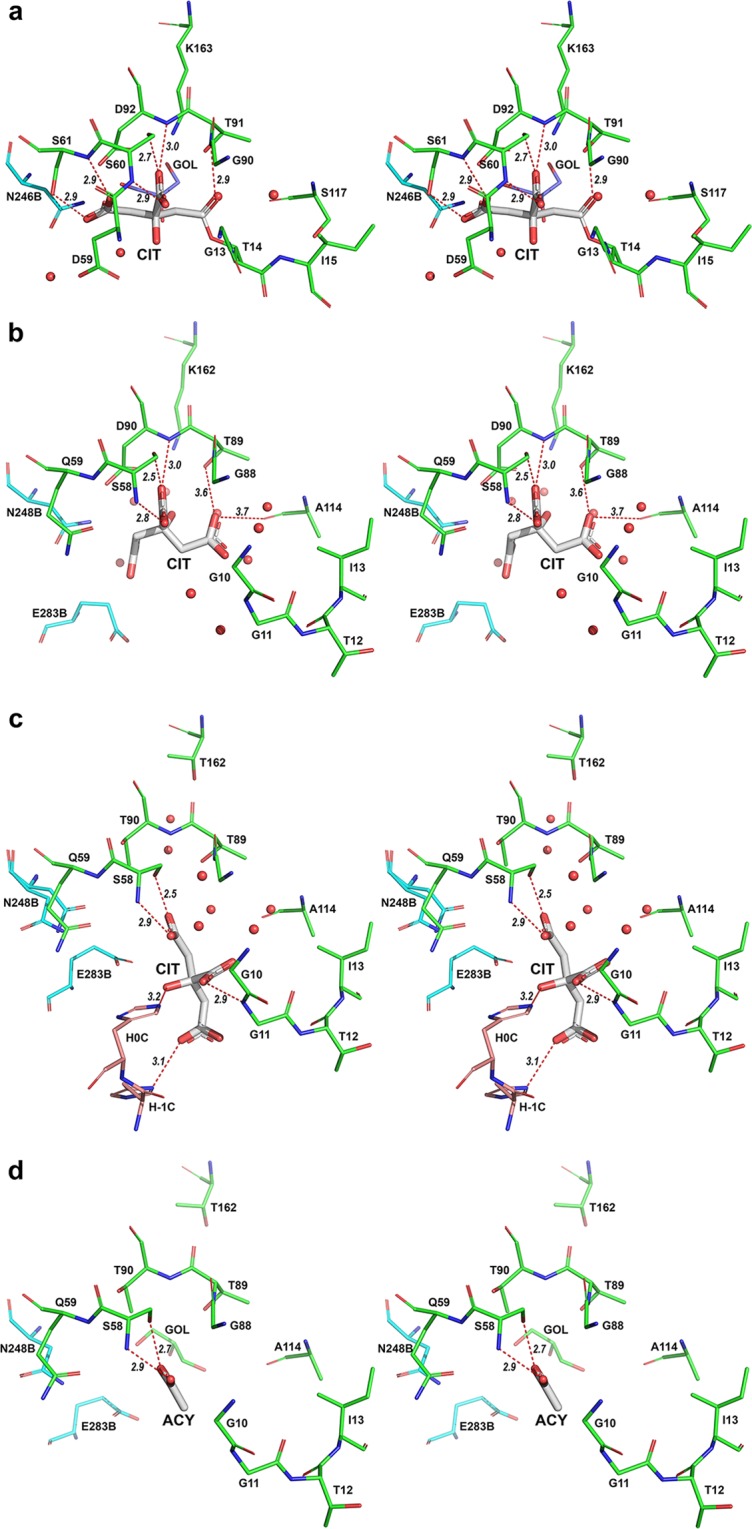
A stereo representation of L-asparaginase active sites occupied by citrate ions. In all panels, residues are shown in stick representation and waters are depicted as red spheres. In each case, the primary ligand is represented by thicker sticks with C-atoms painted gray. Protein residues are shown with C-atoms colored green in the major protomer, cyan in the first minor protomer, and orange in an additional protomer (if present). Significant interactions between the primary ligand and the enzyme are shown as red dashed lines and the distances between interacting atoms are shown in italics. All active site residues are labeled. (**a**) The active site of EcAI (and EcAI(T162A)) with covalently-linked citrate moiety (CIT) and an accessory ligand, glycerol (GOL), observed in crystals grown at pH 4. The panel was prepared based on the structure of monomer A of the EcAI(T162A) but it is representative for all active sites of both EcAI(wt) and EcA(T162A). (**b**) The active site of EcAII(wt) with a citrate anion bound, fund in crystals grown in the presence of 0.17 M ammonium citrate at pH 7. This structure is representative for all four active sites of this complex (PDB code 6nxb) present in the asymmetric unit. (**c**) The active site of the EcAII(D90T,K162T) double mutant with a citrate anion bound, seen in crystals grown in the presence of 0.17 M sodium citrate and 10 mM L-Asn at the pH range 5.3–6.3 (structures EcAII^D90T/K162T^-L-Asn50, EcAII^D90T/K162T^-L-Asn56, and EcAII^D90T/K162T^-L-Asn62). In each of these three structures, the ordered citrate anion was found in only one of the two active sites present in the asymmetric unit (protomer B). Notably, in this case citrate-binding is stabilized by interactions with the His_6_-tag from a symmetry-related molecule. (**d**) The active site of the EcAII(D90T,K162T) double mutant with a citrate anion bound, seen in crystals grown in the presence of 0.17 M ammonium citrate at pH range 5.3–7 in which His_6_-tag is not involved. Such cases are represented by protomer A of three structures mentioned in panel **c**, as well as all four active sites in the structures EcAII^D90T/K162T^-L-Asn7_s_ and EcAII^D90T/K162T^-apo7. In these structures the ordered fragment of the citrate anion was modeled as acetic acid (ACY).

A canonical substrate (L-Asn) and product (L-Asp) (in β-protonated state) are stereochemically compatible with the active sites of either EcAI or EcAII. These ligands form eight well-defined H-bonds with the residues of the enzymes or with conserved structural waters (see Fig. S1). Therefore, it is expected that most of the alternate ligands of L-asparaginases should display significant structural and chemical similarity to a canonical substrate. A citrate anion is a very good example, as illustrated in Fig. 3. In crystals of EcAI (wt or T162A), C5-atom of citrate is covalently bound to Thr14(OG1), forming an acyl-enzyme moiety (Fig. 4a). When compared to the complex of EcAII with L-Asp, formed at pH 5 (Fig. S3a), it is clear that binding modes in both cases are very similar and represent the mimicry illustrated in Fig. 3b. The citrate anion in the EcAI active site participates in six H-bonds with the enzyme molecule, depicted in Fig. 4a. Three of these H-bonds, two contributed by the α-carboxyl group and one due to an interaction between β2-carboxylate and an invariant structural water, are common to structures of both enzymes. Three additional interactions are unique to the EcAI complex, due to differences in amino acid composition of the enzymes, chemical and structural differences of ligands, and a re-orientation of β2 carboxylate of citrate covalently bonded to the Thr14(OG) atom. These interactions include H-bonds of β1-carboxylate oxygens with the N and OG atoms of Ser61 and H-bond between the carbonyl oxygen of β2 and the Thr91(N) atom. Such interactions agree with the expected protonated state of β1-carboxylate of the citrate ligand at pH 4–4.3. In general, when compared to the structure of the canonical complex, a citrate anion is well accommodated and stabilized in the active site of EcAI.

In the EcAII(wt) complex (PDB ID 6nxb), a citrate anion occupies a site equivalent to the one described above for EcAI, but orientations of the ligands are different in both structures (Fig. S3b). A relationship between the citrate and L-Asp in the canonical complex is illustrated in Fig. 3c. It is interesting to consider why neither β1 nor β2 carboxylate of citrate occupies the site filled by the side chain of the canonical ligand. The EcAII^wt^-apo7 complex is formed at pH 7.0, i.e. with all three carboxylates predominantly in a charged state. Mimicking the side chain in the canonical complex by any β-carboxylates would result in repulsive interactions with the carbonyl oxygen of Ala114. On the other hand, in the observed conformation, the O1 atom of β1-carboxylate forms two H-bonds with Thr89(OG1) and with an invariant structural water. The latter interaction, as well as three additional H-bonds contributed by α-carboxylate of citrate, are shared with the canonical complex.

In early reports on kinetics of hydrolysis of various analogs of L-Asn by EcAII, their authors indicated the central role of the α-carboxylate for binding affinity^20,21^. By comparing the canonical complex with the two citrate complexes described above it is clear that only the three H-bonds contributed by the α-carboxylate are common to all of them. This observation further emphasizes the significance of this group in binding to an active site of L-asparaginase by ligands that are structurally and chemically more distant from a canonical substrate. Additionally, a previously described correlation between the role of L-Asp as a product, substrate, or inhibitor of EcAII and pH^17^ is well illustrated here, when conformations of β1 (EcAII-citrate at pH 7), β2 (EcAI-citrate pH 4), and the side chain of L-Asp (3eca) are compared. This comparison points towards the interaction between the carboxylate and the carbonyl oxygen of Ala114_EcAII_ (or Ser117_EcAI_) as being stabilizing (L-Asp – substrate/inhibitor) or destabilizing (L-Asp – product), depending on the protonation state.

Two other examples illustrate interactions of the citrate anion with the active site of the double mutant EcAII^D90T,K162T^. Both Asp90 and Lys162 are invariant in all L-asparaginases, including type I and II enzymes from mesophilic bacteria^8^ as well as thermophilic asparaginases^22^. These residues are indispensable to catalytic properties of these enzymes^23^, and thus this mutant represents a catalytically “dead” enzyme. However, in contrast to Lys162, Asp90 was shown to be critical for binding of the canonical substrates^24^. The structures of EcAII(D90E) in three different crystal forms, all ligand-free, have been published previously^25^, leaving the role of Asp90 in binding a substrate/ligand molecule still not fully explained.

Three structures of EcA^D90T,K162T^ were determined for crystals grown at pH 5.0, 5.6, and 6.2 in the presence of 0.17 M of citrate anions and 10 mM of L-Asn. Under these conditions, a citrate anion is partly (pH 5.6 and 6.2) to predominantly (pH 5.0) mono-protonated on β-carboxylate, with a significant contribution of the doubly-(β1/β2)-protonated form at pH 5.0. In these crystals two protomers of the enzyme are present in the asymmetric unit. Despite of varying pH of the crystallization solution, all three structures are virtually identical. The structure of the active site in the major protomer B is shown in Fig. 4c. Difference mFo-DFc electron density maps indicated clearly the presence of a citrate anion in this site (Fig. S2c). However, in contrast to the examples discussed above, orientation of the citrate anion does not mimic closely the canonical substrate. Here, the ligand is turned away by ~90° from the active site face defined by residues Thr89-Asp90-Lys162 and it is stabilized by a novel set of interations with the enzyme (Fig. 4c). In fact, two of these interactions are formed with the engineered His_6_-tag contributed by a symmetry-related protomer. Although three conserved interactions described earlier for the α-carboxylate group of citrate are not preserved, this moiety still forms two H-bonds with Ser58, indicating importance of this residue for ligand binding. A serendipitous additional interaction with the main-chain N of Gly11 (Fig. 4c), provides additional stabilization.

Figure 4d shows the active sites formed by the major protomer A in these three structures. It is also representative for all active sites present in the structures of EcAII^D90T/K162T^-L-Asn7_S_ and EcAII^D90T/K162T^-apo7. The latter two structures were determined for crystals grown at pH 7, with the citrate anion triply-charged. Difference mFo-DFc electron density maps contained a triangular peak near the side chain of Ser58(OG), clearly associated with a moiety larger than a water molecule (Fig. S2e). Location of this peak matches well the position of α-carboxylate of the citrate anion found in protomers B. This peak was modeled as acetic acid, meant to mimic part of the citrate anion (Fig. 4d).

### Mass spectrometry studies of citrate binding to EcAII

Not having access to EcAI, we used preparations of EcAII to test whether citrate binding in the active site of the enzyme under pH conditions close to those reported in the crystal structures of EcAI could be shown using mass spectrometry (MS). We considered such a change to be justified by the extensive structural similarity of the EcAI and EcAII active sites (Fig. S4), and the functional equivalence of both enzymes. We conducted MS experiments under conditions of low pH, with and without 200 mM citrate and/or 25 mM L-Asp. The results are shown in Fig. S5. The measured molecular weight of the enzyme (35,659.0) agrees with the theoretical value (35,660.0) to within 1 dalton (Fig. S5A). In the presence of 200 mM citrate at pH 4.0 we found a second peak (38.1% abundance) with the molecular weight higher by 174.0, which corresponds to the covalent addition of a citrate molecule (MW = 192.1) associated with removal of a water molecule (Fig. S5B). MS spectrum for EcAII in a presence of 25 mM L-Asp and 50 mM acetate buffer at pH 4.0 showed only the unmodified enzyme (Fig. S5C), whereas combination of 200 mM citrate and 25 mM L-Asp resulted in covalent binding of citrate only (35.1% abundance, Fig. S5D). These results provide additional support for the hypothesis that the structure of EcAI, as re-refined here, is correct.

## Discussion

Evolution of L-asparaginases resulted in highly optimized shapes and compositions of their active sites, enabling binding and hydrolysis of the canonical substrates L-Asn and L-Gln (and β-protonated L-Asp and L-Glu). However, the presence of charged and hydrophilic residues in the active site pockets of these enzymes allows binding of other ligands, such as small amino acids, succinates, maleates, and derivatives thereof. Whereas the binding affinities of such ligands are lower compared to canonical substrates, these molecules are present in physiological fluids and their cumulative contribution may affect the catalytic activity of L-asparaginase. In fact, as illustrated here by the example of a covalent complex between EcAI and a citrate anion, some of these opportunistic ligands may serve as viable substrates for this enzyme. Citric acid (or citrate), significantly bulkier than L-Asn or L-Gln, is not an obvious ligand to be found in the highly specialized active site of L-asparaginase. Yet, as we demonstrated here based on the reinterpreted structures of EcAI and the new structures of EcAII, under some conditions a citrate ion can be accommodated into the active site cavity and even displace the canonical substrate or product. Cumulatively, the examples presented here support the need for cautious analysis of crystallographic experimental data that may lead to very divergent results and conclusions, even in the case of a well-studied enzyme.

The covalent ligand of EcAI was described by Yun *et al*. as an acyl-enzyme intermediate resulting from a nucleophilic attack by Thr14 on the carbon CG of L-Asn. However, the large electron density for the ligand must have represented more than a single L-Asn molecule. It was interpreted by the authors as a composite of an ester of Thr14:L-Asp and a molecule of L-Asn, non-covalently bound in an opposite orientation. Such a binding mode of L-Asn has never been reported previously. The authors assumed half occupancy for L-Asn and L-Asp, resulting in a composite ligand, in which some atom sites are only half occupied (i.e. OG1 of Thr14, CG and CB of L-Asp and N, CG, OD1, and ND2 of L-Asn). Close inspection of the structures reported by Yun *et al*. prompted us to re-analyze that earlier report, resulting in re-refinement of their structures and re-interpretation of the results.

Yun *et al*. did consider a possibility that the electron density contributed by the active site ligand molecule(s) might originate from binding a citrate anion but rejected it. We found their reasoning unconvincing for several reasons. The first one being that the results could be interpreted more simply. We found that the mFo-DFc electron density map based on their PDB entry 2him had a number of significant peaks in and near the site of ligand(s) that were not modeled (Figs 1d and 2). Also, ADPs for half-occupied atom sites were unusually low when compared to their direct environment formed by the enzyme (Fig. 2). Based on the description of the protocol that led to the growth of crystals and diffraction data collection, we found it questionable whether any L-Asn could still be present in the solution and/or crystals by the time diffraction data were measured. Purified enzyme was dissolved in Tris-HCl buffer at pH 7.5 and a putative complex with L-Asn was formed by dissolving solid L-Asn in a protein solution and subsequent removal of an excess of undissolved ligand by centrifugation. The resulting complex was later mixed at the 1:1 ratio with the crystallization buffer containing 0.1 M citric acid/trisodium citrate (pH 4.0) and 1 M sodium chloride. Based on the rate of catalytic activity of EcAI, we predict that most if not all the L-Asn substrate has been already hydrolyzed by the enzyme.

In their interpretation, Yun at al, stated “However, [the electron density] was consistently too large for either asparagine (substrate) or aspartate (product) and was interpreted as a mixture of a productive conformation of aspartate and an unproductive, alternative conformation of asparagine”^13^. Apart from describing the ligand as L-Asp, whereas the observed covalent adduct would be an acyl-enzyme intermediate, the authors invoked four reasons for their claim that the observed ligand is not citrate. First, they stated that “the side-chain electron density is clearly asymmetric and consistent with the larger oxygen and smaller nitrogen of L-Asn and not the symmetric carboxylate of citrate”. This must refer to the interaction of these two atoms with the N and OG1 atoms of Ser61, respectively. However, the ADPs of the half-occupied O and N atoms of L-Asn are significantly lower than for the interacting atoms of Ser61, while an opposite effect would normally be expected. We see no corresponding asymmetry in our final electron density maps, the ADPs of both oxygens of the carboxylate of the citrate are comparable, and each is about 50% higher than the ADPs of the respective hydrogen bonded atoms of Ser61. The ADPs of the α-carboxylate atoms of citrate in the re-refined structure are also comparable to the ADPs of the interacting N and OG of Ser60, as well as N of Thr93. Since, as indicated by Yun *et al*., it is not possible at this resolution to unequivocally differentiate between N and O atoms based only on the electron density, and since both H-bonded partners of the “L-Asn” side chain are potential donors, we consider the re-interpreted structure to be more likely than the original one.

The second argument is that “Citrate, having a carboxylic acid groups at either end, would not be expected to react with a nucleophilic threonine”. This argument contradicts the published data showing, through ^18^O exchange, that L-Asp is actually a substrate of L-asparaginase^17^. The latter authors also demonstrated that the reaction with L-Asp is promoted by low pH, when β-carboxylate is in the protonated state. An analogous scenario could take place in the case of citrate under acidic conditions (in this case, pH around 4.0).

We cannot comment on the third argument, namely that individual densities for the ligands were seen in the T14V and T14A mutants of EcAI, since these data were not shown. However, the fourth argument, namely that “the enzyme was about two-fold more active in a citrate buffer at pH 6.0 than in a bis-Tris buffer at the same pH (data not shown)” does not really prove that a bound citrate would act as an inhibitor of the enzyme. The inhibition data refer to pH 6.0, while, as indicated above, the pH of the crystallization solution was near 4, where activity of L-asparaginase is already marginal.

Under the crystallization conditions (pH ~4) both the β1 and β2 carboxyl groups of citrate are predominantly protonated (Fig. 3d). The binding mode of such a citrate anion to the active site of EcAI mimics very closely the binding of a canonical substrate (Fig. S3a). At pH 7.0 a majority of citrate anions are fully charged, suggesting that under such conditions the canonical binding may not be preferable. Such prediction is exemplified by the structure of EcAII-citrate complex (EcAII^wt^-apo7). In that case only the α-carboxyl of the citrate occupies the location observed in a canonical complex, whereas neither of the β-carboxylates assumes a position of the side chain in a canonical complex. In EcAII^wt^-apo7, a fragment of the ligand contributing β2-carboxylate approximates somewhat the side chain of a canonical substrate; however, in the observed conformation it prevents Thr12 from assuming the closed conformation.

We predict that binding of citrate to EcAII is significantly weaker under comparable conditions than to EcAI, due to the differences between the sizes and compositions of the active site pockets. One of the residues with a side chain projecting towards the active side of EcAI is Ser61, but its equivalent in EcAII is bulkier Gln59. More importantly, Glu283, contributed to the active site of EcAII by the minor protomer, does not have an equivalent in EcAI. The presence of Glu283 not only reduces the size of the active site cavity, but its charged carboxyl group further destabilizes binding of a negatively-charged ligand such as citrate. As a result, both the β1-carboxylate of citrate and the side chain of Glu283 assume conformations that maximize their separation.

The remaining five structures of EcAII describe its inactive variant with both Asp90 and Lys162 replaced by threonines. It was previously reported that mutation of Asp90 greatly impairs binding properties of EcAII^24^ and the structures of the D90E mutant were also published^25^. Here, three isomorphous structures represent crystals grown in the presence of 10 mM L-Asn and 0.17 M citrate at the pH range 5.0–6.2. In all three structures one of the two active sites is occupied by a citrate anion in a conformation different from the two examples described earlier. This conformation, however, being attributed to two interactions with the engineered N-terminal His_6_ affinity tag from a symmetry-related protomer, is an experimental artifact. Interestingly, even in the case of these complexes, the α-carboxylate of the ligand occupies the same site as the equivalent group of the canonical ligand, emphasizing the central role of the α-carboxyl group of any ligand in binding to L-asparaginase active site.

The remaining active sites, one in each of the three structures, appear not to be occupied by stably bound ligand molecules other than solvent. Their state appears to be the same as in the structures of EcAII^D90T/K162T^-apo7 and EcAII^D90T/K162T^-L-Asn7s, the former obtained at pH 7 in the absence of L-Asn, and the latter after subsequent soak in 10 mM L-Asn. However, a common feature of these active sites is a triangular blob of electron density located near the side chain of Ser58, i.e. in the site of α-carboxylate of substrate in the canonical complex. As mentioned earlier, very likely this site serves as the initial anchor for the ligand. It seems plausible that a ligand is present in the “unoccupied” active sites, but only its α-carboxyl group is ordered.

The examples of an opportunistic binding of a ligand to the active site L-asparaginase, presented in this report, clearly illustrate the need for critical interpretation of experimental crystallographic data. We postulate that the unusual binding mode of L-Asn in the active site of EcAI^13^ is not supported by the previously published structure.

The structure of the covalent complex between EcAI and citrate is only the second example of such an intermediate of L-asparaginases, following previous observation in a variant of EcAII^15^. Both structures are potentially useful for the understanding of the still incompletely described mechanism of enzymatic reaction. If confirmed by additional experiments, the presence of such intermediates would provide support of the double-displacement mechanism, with the threonine side chain (Thr14 or Thr12 in EcAI/EcAII, respectively) acting as the primary nucleophile. Such a mechanism was recently criticized^26,27^ on the basis that the observed acyl-enzyme intermediates could be artefacts of either crystallization conditions or the use of mutants. In light of the results described by Yun *et al*.^13^ and their re-interpretation presented here, the second argument appears to be questionable, since a covalent intermediate with Thr14 (nucleophile) is formed by the EcAI(wt). However, full understanding of the enzymatic mechanism of L-asparaginase still awaits its clarification.

## Materials and Methods

### Preparation of mutated EcAII samples

Preparation of plasmids and bacterial cell lines used for expression was described previously^28,29^. The identity and correctness of all plasmids used for heterologous expression of EcAII and its double mutant, EcAII^D90T/K162T^, were confirmed by DNA sequencing. Expression experiments were performed using *E. coli* cell line deficient in three endogenous L-asparaginse genes (a triple knockout), thus assuring that all L-asparaginase activity would originate from the recombinant gene (whereas such a procedure is critical for kinetic and/or functional studies, it was not crucial to strictly structural experiments discussed here). In all cases L-asparaginase was secreted to media in a general form Met-(His)_6_-EcAII, and the affinity tag was not excised prior to structural studies. Additional details of the cloning and expression of EcAII are provided in the Supplementary Data.

The purification protocol consisted of two steps, Ni-affinity chromatography in batch mode and size exclusion chromatography (for details see Supplementary Data). On average about 15–20 mg of purified enzyme could be recovered from one liter of *E. coli* cell culture. It is worth noting that Ni-affinity chromatography resulted in high-purity preparations (as monitored by SDS-PAGE); however, subsequent gel-filtration contributed to (i) buffer replacement, (ii) increased purity, and (iii) lowered content of aggregates, all of which are significant for successful crystallization.

### Mass spectrometry experiments

Mass spectrometry data were acquired on an Agilent 6100 Series Quadrupole LC/MS System, (Agilent Technologies, Inc., Santa Clara, CA) equipped with electrospray source, operated in the positive-ion mode. Separation was performed on Zorbax 300SB-C3 Poroshell column (2.1 mm × 75 mm; particle size 5 μm). Mass spectra were recorded across the range 300–2000 *m*/z. The UV signal was collected at 280 nm with a reference at 360 nm. Data acquisition and analysis were performed using OpenLAB CDS ChemStation Edition C.01.05.

### Crystallization, data collection, structure solution, and refinement of the new EcAII structures

For crystallization, protein solution (15–18 mg/ml) in 50 mM HEPES buffer (pH 7.0) and 200 mM sodium chloride was mixed with the equivalent volume of precipitation buffer containing 17–18% (w/v) PEG3350, 0.17–0.18 M citrate buffer at appropriate pH, and (in selected experiments) 10 mM L-Asn. Crystals denoted EcAII^D90T/K162T^-apo7 and EcAII^wt^-apo7 were not exposed to L-Asn prior to X-ray data collection, whereas crystals of the EcAII^D90T/K162T^-L-Asn7_s_ complex were soaked prior to freezing for several minutes in the above-mentioned solution enriched with 10 mM L-Asn. In the case of crystals EcAII^D90T/K162T^-L-Asn50, EcAII^D90T/K162T^-L-Asn56, and EcAII^D90T/K162T^-L-Asn62, precipitation buffer also contained 10 mM L-Asn. Crystals used for the X-ray data collection were transferred to the cryo-protecting solution consisting of the precipitation buffer with either the concentration of PEG3350 increased to 40% (w/v) or with addition of 25% (v/v) glycerol. Despite several attempts, crystals of the EcAII^D90T/K162T^ variant grown in the presence of L-Asn could not be successfully frozen for X-ray data collection, with the procedure leading to very poor diffraction and dramatically increased mosaicity. Thus, diffraction data in these cases were collected at room temperature (20 °C) from crystals mounted in quartz capillaries. Whereas the initial diffraction images for the latter crystals were of high quality, as evidenced by sharp spots and low mosaicity, radiation damage was evident throughout the course of experiments, limiting the number of useful images that could be included in subsequent processing. As a result, the multiplicity of reflection measurements was somewhat reduced; however, the final datasets were still quite complete (>95%) and the quality of the resulting electron density maps was satisfactory. The final statistics of data processing are presented in Table 2.

Diffraction experiments for all the EcAII crystals were performed using an in-house conventional X-ray source, a Rigaku rotating anode MicroMax-007HF generator operated at 40 kV and 30 mA, with the CuKα wavelength of 1.5418 Å. Images were recorded in a continuous mode with a Dectris Eiger 4 M pixel detector. The experimental images were processed using the HKL3000 suite^30^. Whereas utilization of synchrotron radiation would have unquestionably resulted in higher resolution X-ray data, we are confident that all biologically-relevant and most of structurally-relevant questions related to the structures described here can be satisfactorily addressed based on the acquired experimental data.

The initial phases for these structures were obtained either by molecular replacement with the program Phaser^31^ and the structure of EcAII monomer (PDB ID 3eca)^14^ used as a search model, or by directly phasing data with a previously determined isomorphous structure. After either procedure, following rigid-body refinement with Refmac5^32^, all structures were subjected to several cycles of more elaborate refinement with the same program, interspersed with visual inspection and manual corrections with Coot^33^. The near-final models were evaluated by the MolProbity server^18^ and completed by applying additional corrections coupled with structural refinement. The statistics for the final structural models are shown in Table 3.

**Table 3:** Structure refinement of the EcAII data sets.

	EcAII^D90T/K162T^-L-Asn50	EcAII^D90T/K162T^-L-Asn56	EcAII^D90T/K162T^-L-Asn62
Resolution range (Å)	2.15–40.0 (2.15–2.21)*	2.15–40.0 (2.15–2.21)	1.85–40.0 (1.85–1.90)
Completeness (%)	92.6 (78.8)	98.4 (94.0)	97.0 (76.2)
σ-cutoff	none	none	none
No. of reflections, working set	41,565 (2444)	44,290 (2926)	68,407 (3804)
No. of reflections, test set	2017 (139)	2205 (151)	2047 (112)
Final R_cryst_	0.129 (0.204)	0.135 (0.216)	0.130 (0.233)
Final R_free_	0.170 (0.263)	0.183 (0.250)	0.156 (0.272)
ADP parametrization	isotropic	isotropic	isotropic
No. of non-H atoms			
Protein	4729	4727	4779
Ions/ligands	17	17	17
Water	293	314	424
Total	5039	5058	5220
R.m.s. deviations from targets			
Bonds (Å)	0.02	0.02	0.019
Angles (°)	2.00	1.97	1.94
Average B-factors (Å^2^)			
Protein	32	32.3	25.5
Water	35.5	37.3	36.2
Ions/ligands	53.4	51.7	43.3
Ramachandran statistics (%)^†^			
Favored/outliers	98.5/0.2	98.3/0.3	98.5/0
Clashscore/percentile^†^	1.28/100^th^	0.95/100^th^	1.27/100^th^
MolProbity score/percentile^†^	0.99/100^th^	1.06/100^th^	0.85/100^th^
PDB accession code	6nx6	6nx7	6nx8
	**EcAII**^**wt**^**-apo7**	**EcAII**^**D90T/K162T**^**-L-Asn7**_**S**_	**EcAII**^**D90T/K162T**^**-apo7**
Resolution range (Å)	1.75–22.85 (1.75–1.80)	1.97–26.55 (1.97–2.02)	1.93–26.53 (1.93–1.98)
Completeness (%)	97.7 (92.6)	96.3 (89.1)	96.0 (81.2)
σ-cutoff	none	none	none
No. of reflections, working set	115,868 (7820)	80,972 (5327)	85,704 (5222)
No. of reflections, test set	2992 (216)	2654 (177)	2649 (130)
Final R_cryst_	0.142 (0.213)	0.162 (0.270)	0.155 (0.227)
Final R_free_	0.192 (0.281)	0.226 (0.353)	0.224 (0.300)
ADP parametrization	isotropic	isotropic	isotropic
No. of non-H atoms			
Protein	9657	9441	9440
Ions/ligands	75	29	30
Water	1316	1049	1184
Total	11,044	10,059	10,658
R.m.s. deviations from target values			
Bonds (Å)	0.02	0.02	0.02
Angles (°)	2.21	2.29	2.32
Average B-factors (Å^2^)			
Protein	31.1	27.2	35.9
Water	37.5	34.1	43.1
Ions/ligands	47.6	40.3	53.9
Ramachandran statistics (%)			
Favored/outliers	98.2/0	97.8/0.1	97.7/0.1
Clashscore/percentile	1.83/99^th^	2.33/99^th^	3.44/99^th^
MolProbity score/percentile	0.94/100^th^	1.27/99^th^	1.28/99^th^
PDB accession code	6nxb	6nx9	6nxa

^†^Values calculated with the program MolProbity (http://molprobity.biochem.duke.edu/)^18^.

*Values shown in parentheses correspond to the highest resolution shell.

### Re-refinement of the previously published structures of EcAI

Two structures of EcAI crystallized in the presence of citrate were previously deposited in the PDB (PDB ID 2him and 2p2n)^13^. Crystals of EcAI(wt) and its T162A mutant are isomorphous, but the data set collected from the latter one extends to higher resolution and the 2mFo-DFc map downloaded from the PDB showed similar density for the ligand bound to all four molecules in the asymmetric unit. In the case of the mutant, the map also strongly suggested that the ligand molecules are covalently bound to the OG1 atom of Thr14, but the identity of the ligand remained to be proven. The electron density map in the regions of the active sites is less clear for the native EcAI. The PDB-deposited model (2p2n) includes Thr14 in only one molecule, although the electron density for the ligands themselves is comparable in all four active sites.

For re-inspection of both entries, we used the deposited experimental X-ray intensities and the atomic coordinates (after removing the molecules of ligands and solvent) as starting models. In the case of the native structure (2p2n), in order to minimize potential bias, we subjected the starting model to the “rebuilding” protocol using the program MR-Rosetta^34^, and followed with refinement interspersed by manual corrections using the programs Refmac5 and Coot. In parallel, we simply refined the starting model after manual corrections. Since the results of both approaches were virtually identical, only those resulting from the latter one are discussed here. The starting model for the T162A mutant was directly refined with Refmac5 and rebuilt with Coot. Additionally, although the original refinement of the latter structure (2him) utilized data extending to 1.82 Å resolution, we found that the deposited structure amplitudes extended to 1.74 Å. While the results of refinement at either resolution were comparable, we considered addition of ~12,000 reflections beyond the originally reported resolution limit to be justified.

### Accession codes

The coordinates and structure factors have been deposited in the Protein Data Bank with the accession numbers 6nxc and 6nxd (EcAI), and 6nx6, 6nx7, 6nx8, 6nx9, 6nxa, and 6nxb (EcAII).

## Supplementary information

Supplementary material
