# Supplementary material for: Opportunistic complexes of E. coli L-asparaginases with citrate anions
Source: Sci Rep. 2019 Jul 30;9:11070. doi: 10.1038/s41598-019-46432-0 (PMC6667453; doi:10.1038/s41598-019-46432-0)
Supplement: Supplementary file 1 — Supplementary material [file 41598_2019_46432_MOESM1_ESM.pdf]

Supplementary Data

Opportunistic complexes of *E. coli* L-asparaginases with citrate anions

Jacek Lubkowski[1*], Wai-Kin Chan[2] and Alexander Wlodawer[1]

[1]Macromolecular Crystallography Laboratory, Center for Cancer Research, National Cancer Institute, Frederick, MD 21702, USA.

[2]Department of Bioinformatics and Computational Biology and The Proteomics and Metabolomics Core Facility, The University of Texas MD Anderson Cancer Center, Houston, TX 77030, USA.

[*]Corresponding author:
E-mail: lubkowsj@mail.nih.gov
Phone: +1-301-846-5494

Preparation of the samples of mutated EcAII

The DNA sequence encoding the EcAII gene with N-terminal $(His)_6$-sequence (without a linker) was cloned into the commercial pET22b(+) vector as described earlier[1]. This vector introduces a secretion sequence pelB leader into the resulting open reading frame. After amplification and purification, the vector was transformed into the *E. coli* JC2 strain (in this strain, originated from BL21 (DE3), three genes *ansA*, *ansB*, and *iaaA* encoding L-asparaginase I, L-asparaginase II, and isoaspartyl aminopeptidase, respectively, were deleted[2]), using standard protocols and monoclonal cultures were grown on agar plates supplied with ampicillin. Subsequently, small cultures of transformed cells were grown for generation of a glycerol stock or as starter cultures for large-scale expression.

An overnight culture of transformed cells was grown at 37 °C in the presence of ampicillin. This culture was subsequently used to inoculate large-scale cultures (the dilution factor 1:50). Inoculated, large-scale cultures were cultivated at 37 °C in the presence of ampicillin until the optical density (at 600 nm) reached 0.7-0.9 (usually 2-3 hours). At this point protein expression was induced by addition of isopropyl β-D-1-thiogalactopyranoside (IPTG) to the final concentration of 1 μM. Induced cells were cultured overnight (16-18 hours) in the shaker-incubator at 37 °C and agitation rate of 225 rpm. On the following morning, cells were separated by centrifugation at 6000x*g* for 15 min and filtered through 0.45 μm filter. The aliquot of freshly prepared PMSF was added to the supernatant at the final concentration of 0.5 mM, in addition to phosphate buffer (50 mM, pH 7.4) and sodium chloride (0.3 M). The EcAII-containing media were then mixed with a Ni-affinity resin (His60 Ni Superflow resin, Clontech) and placed on a rocker for 3 hours at 5 °C. The resin was isolated on a gravity column and subjected to series of washing steps as recommended by the supplier. Protein was eluted with 0.5 M imidazole. The eluate from Ni-affinity resin was concentrated and applied to the size-exclusion, S200 HP column (GE Life Sciences) equilibrated with the buffer composed of 50 mM HEPES pH 7.0 and 200 mM sodium chloride. Finally, purified protein solution was concentrated to 10-20 mg/ml and either used for crystallization experiments or frozen at -80 °C for future use.

Supplementary Table S1. Primers sequences used for generating L-asparaginase double mutant $EcAII^{D90T/K162T}$

Primer No	Mutation	Sequence (5'→3')
Primer 295	D90T	GTCATTACCCACGGTACCDCAACGATGGAAGAAACTG
Primer 296	D90T	CAGTTTCTTCCATCGTTGHGGTACCGTGGGTAATGAC
Primer 316	K162T	GATGGCCGTGACGTCACCACAACCAACACCACCGACGTA
Primer 317	K162T	TACGTCGGTGGTGTTGGTTGTGGTGACGTCACGGCCATC

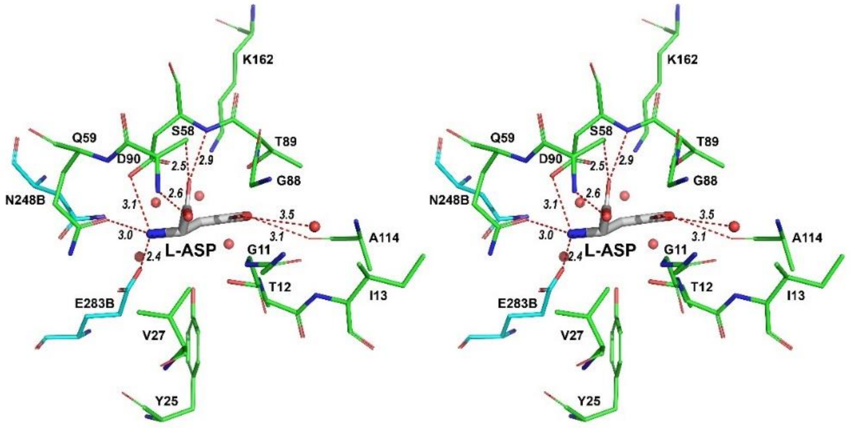

Figure S1. This stereo image is based on the PDB entry 3eca (monomer A) and depicts the active site of EcAII(wt) in complex with L-Asp at pH 5.0. At this pH, the side chain carboxylate is protonated in a significant population of L-Asp molecules. All residues are shown in the stick representation and waters are depicted as red spheres. Ligand molecule is represented by thicker sticks with carbon atoms gray. Protein residues are shown with carbon atoms green in the major protomer and cyan in the minor on, since both contribute to creation of the active site. Eight most significant interactions that define binding and stabilization of L-Asp in the active site are indicated by red dashed lines with interatomic distances (Å) shown in italics. All active site residues are labeled. Since β-protonated L-Asp represents both the product and the substrate (the latter in a catalytic reaction of the oxygen exchange involving the side chain of L-Asp[3]), this figure represents canonical binding of the substrate and/or product to the active site of L-asparaginase and may serve as a reference during analysis of other ligands.

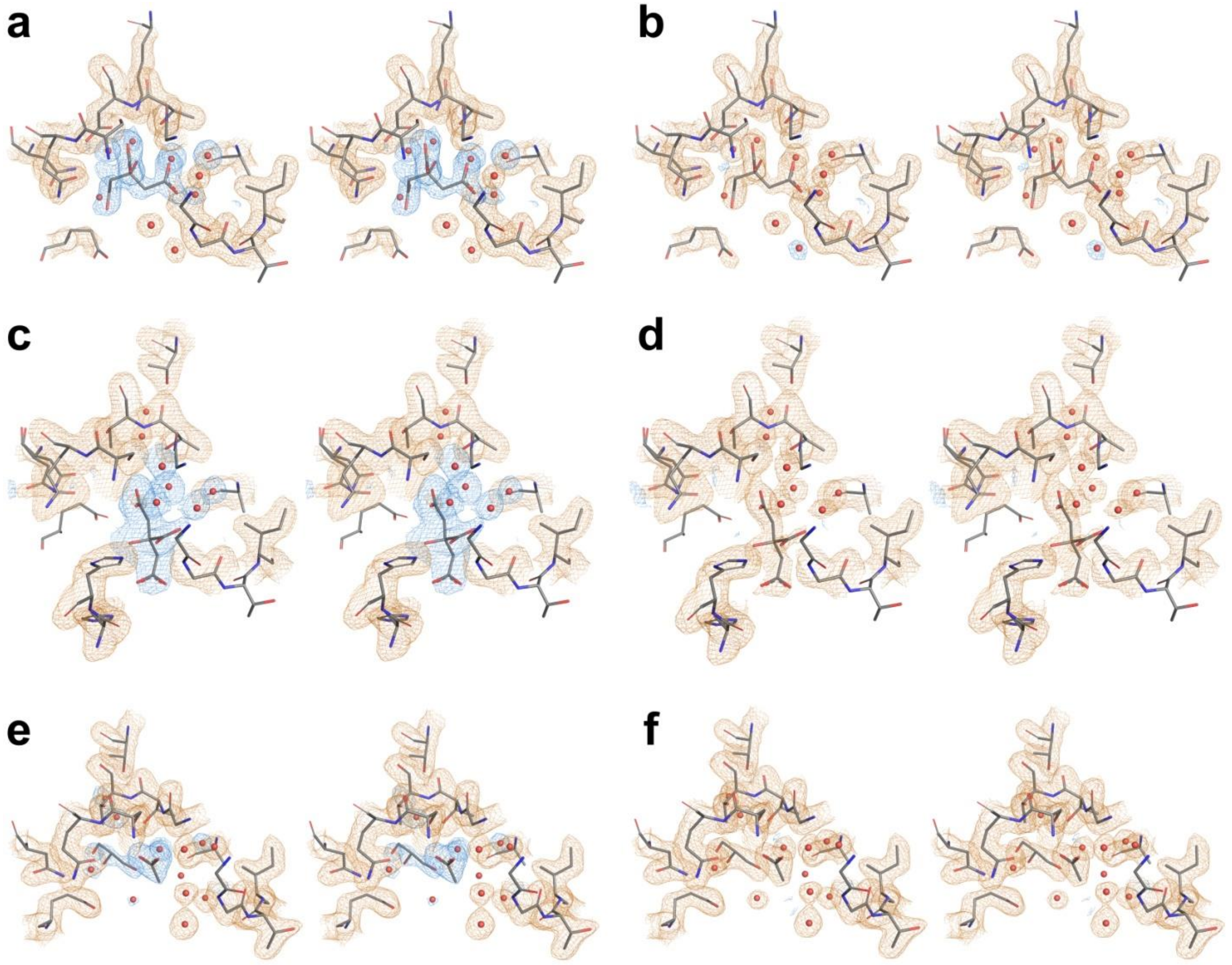

Figure S2. Stereo images of the difference electron density maps, $2mF_o$-DF_c shown in orange and contoured at $1\cdot\sigma$ level and mFo-DFc (blue), contoured at $3\cdot\sigma$ level. No mFo-DFc density peaks are present in these maps at the $-3\cdot\sigma$ level. Panels **a** and **b** represent the active site of $EcAII^{wt}$-apo7, prior to modeling the content of the active site and after final refinement, respectively. Similar pairs of images are shown for $EcAII^{D90T/K162T}$-L-Asn50 (panels **c** and **d**) and $EcAII^{D90T/K162T}$-apo7 (panels **e** and **f**). In the first two structures, mF_o-DF_c electron density peaks (shown in blue) can be easily modeled as citrate anions and a few water molecules and this interpretation is supported by an excellent agreement between the final model and the associated $2mF_o$-DF_c electron density map. In the last case ($EcAII^{D90T/K162T}$-apo7), a triangular blob of mF_o-DF_c electron density, adjacent to the side chain of Ser58, was modeled as an acetate ion, although it is almost certainly associated with the carboxyl group of weakly-bound citrate (or, less likely, L-Asn). In this case, the absence of the side chain of Asp90 (Thr90 in this EcAII variant) creates a void in the active site pocket, which is occupied by the a molecule of glycerol (behind the acetate in panels **e** and **f**).

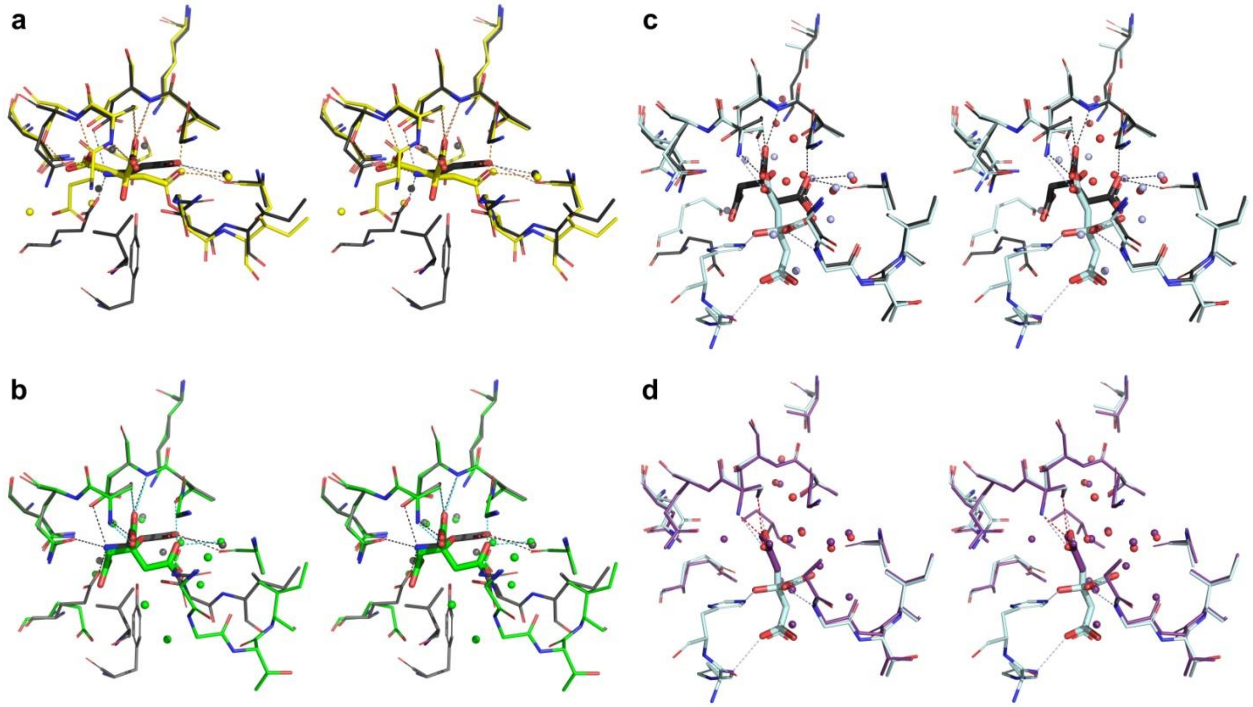

Figure S3. Pairwise comparisons of L-asparaginases active sites bearing citrate anions are illustrated in stereo representation. In case of EcAI, C-atoms are painted in yellow (panels **a** and **b**), in EcAII(wt) C-atoms are colored dark-gray (panels **a** and **c**) or green (panel **b**), they are colored pale-blue in complex of EcAII(D90T,K162T) with ordered citrate anion (panel **c**) and purple-violet for this mutant in complex with partially disordered citrate anion (panel **d**). Panel **a** illustrates a superposition of the EcAI-citrate complex (PDB code 6nxc) with the EcAII(wt)-aspartate complex (PDB code 3eca), panel **b** depicts overlaid complexes of EcAII(wt)-aspartate (3eca, shown in dark grey) and EcAII(wt)-citrate (PDB code 6nxb, shown in green), in panel **c** complex of EcAII(wt)-citrate (6nxb) is superimposed on EcAII(D90T,K162T)-citrate (PDB code 6nx6, monomer B), and in panel **d** overlay of complexes between citrate anion and 6nx6 (monomer B) or EcAII(D90T,K162T) (PDB code 6nx9) is shown. If not indicated, monomer A from each structure was used for these comparisons.

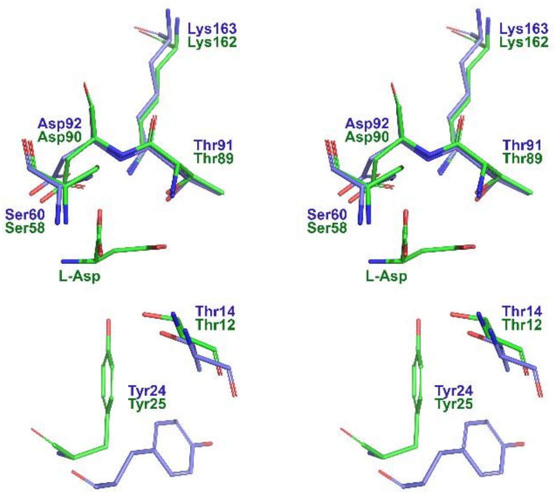

Lys163
Lys162
Asp92
Asp90
Thr91
Thr89
Ser60
Ser58
L-Asp
Thr14
Thr12
Tyr24
Tyr25

Figure S4. Superposition of critical active site residues of EcAI (blue) and EcAII (green). The figure is prepared in stereo representation and the equivalent residues from both enzymes are labeled. A molecule of aspartic acid (the product of catalytic reaction in both enzymes) is shown as determined for the Michaelis complex of EcAII...L-Asp.

A

Deconvolution of Spectrum # 1 @ 2.185 - 2.326 min

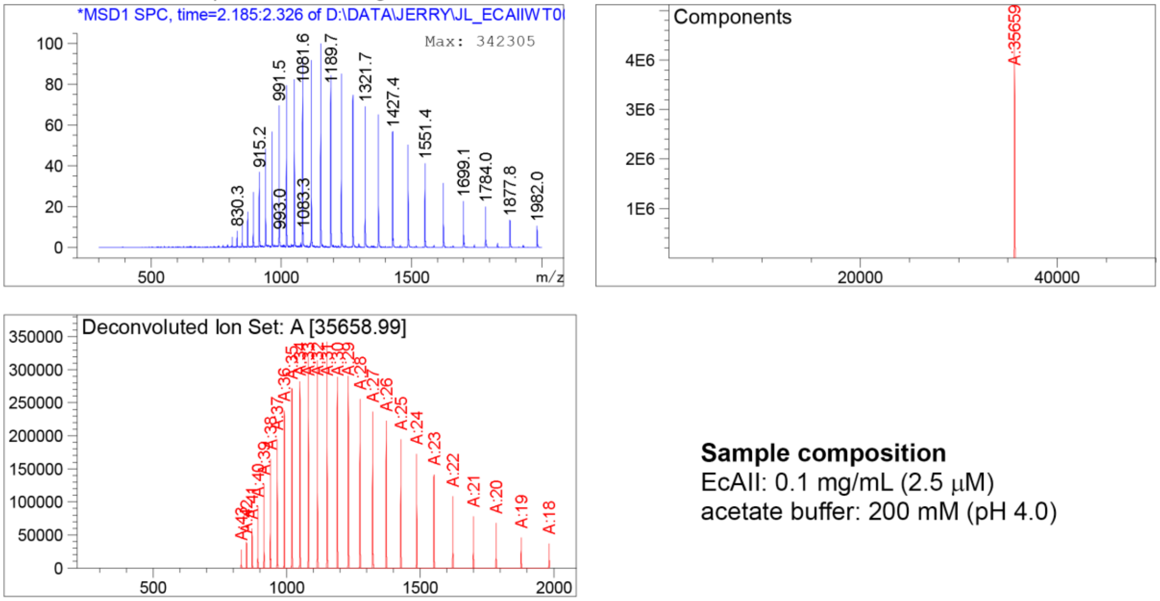

Sample composition
EcAII: 0.1 mg/mL (2.5 μM)
acetate buffer: 200 mM (pH 4.0)

Component	Molecular Weight	Absolute Abundance	Relative Abundance
A	35658.99	4576209	100.00

*** End of Report ***

B

Deconvolution of Spectrum # 1 @ 2.162 - 2.303 min

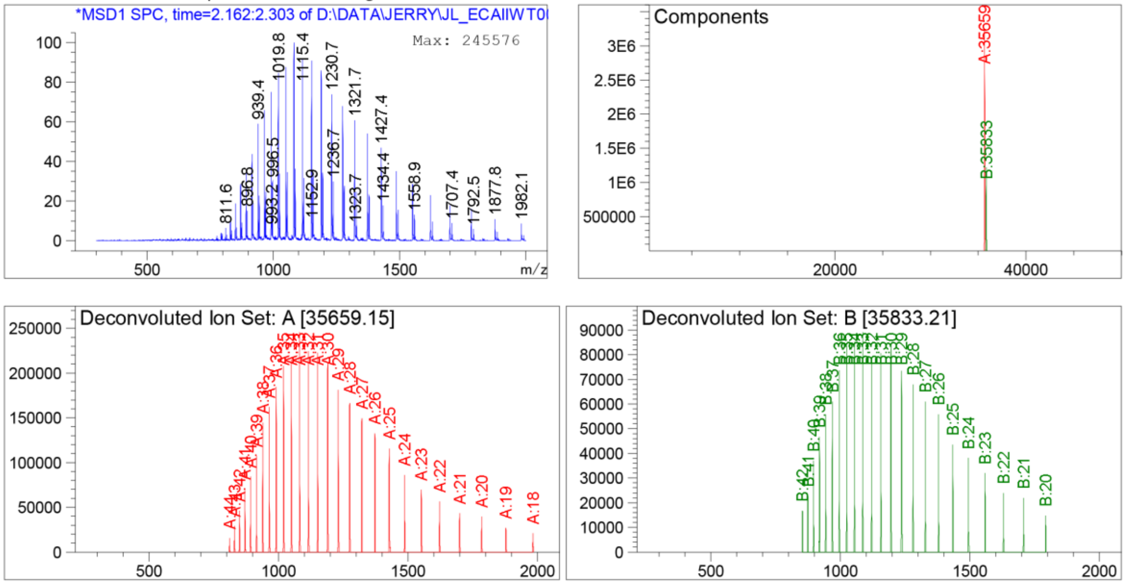

Component	Molecular Weight	Absolute Abundance	Relative Abundance
A	35659.15	3218223	100.00
B	35833.21	1225329	38.07

*** End of Report ***

Sample composition
EcAII: 0.1 mg/mL (2.5 μM)
citrate buffer: 200 mM (pH 4.0)

C

Deconvolution of Spectrum # 1 @ 2.162 - 2.331 min

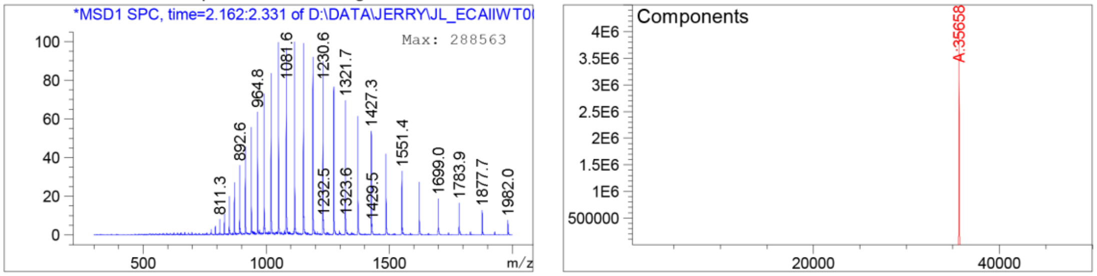

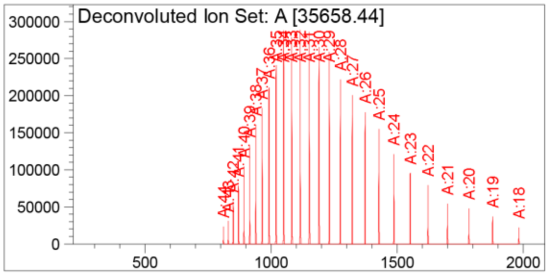

Sample composition
EcAII: 0.1 mg/mL (2.5 μM)
acetate buffer: 50 mM (pH 4.0)
L-Asp: 25 mM

Component	Molecular Weight	Absolute Abundance	Relative Abundance
A	35658.44	4024277	100.00

*** End of Report ***

D

Deconvolution of Spectrum # 1 @ 2.162 - 2.303 min

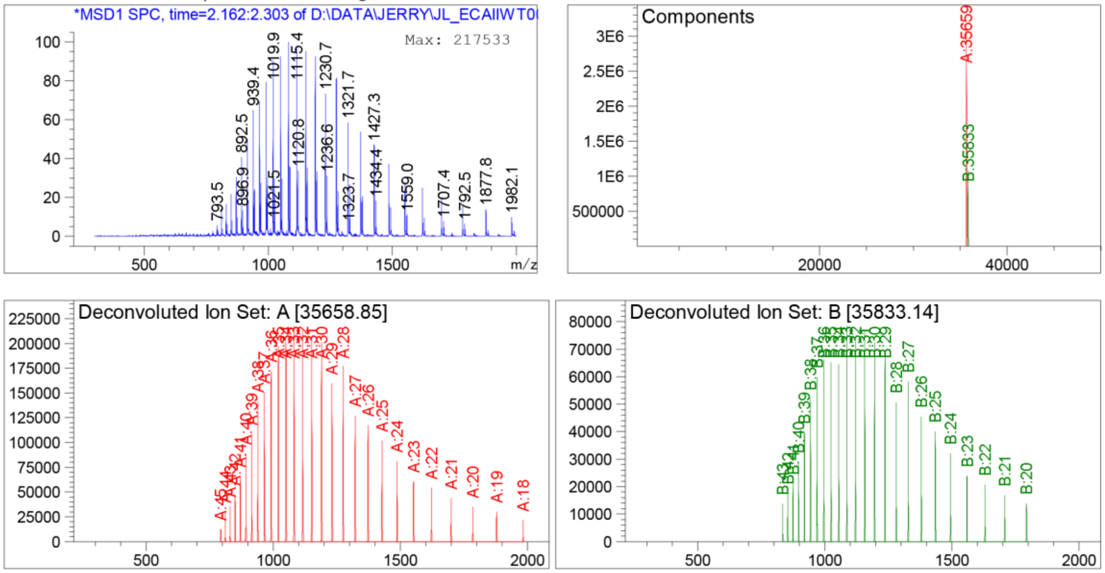

Component	Molecular Weight	Absolute Abundance	Relative Abundance
A	35658.85	3077317	100.00
B	35833.14	1080466	35.11

*** End of Report ***

Sample composition
EcAII: 0.1 mg/mL (2.5 μM)
citrate buffer: 200 mM (pH 4.0)
L-Asp: 25 mM

Figure S5. Results of MS analysis for EcAII (see the main text for details).

References

1. Chan, W. K. *et al.* The glutaminase activity of L-asparaginase is not required for anticancer activity against ASNS-negative cells. *Blood* **123**, 3596-3606, doi:10.1182/blood-2013-10-535112 (2014).
2. Cantor, J. R. *et al.* Therapeutic enzyme deimmunization by combinatorial T-cell epitope removal using neutral drift. *Proc. Natl. Acad. Sci. USA.* **108**, 1272-1277, doi:10.1073/pnas.1014739108 (2011).
3. Röhm, K. H. & Van Etten, R. L. The ^{18}O isotope effect in ^{13}C nuclear magnetic resonance spectroscopy: mechanistic studies on asparaginase from *Escherichia coli*. *Arch. Biochem. Biophys* **244**, 128-136 (1986).
